# Supplementary figures and images for: Transcriptional markers classifying Escherichia coli and Staphylococcus aureus induced sepsis in adults: A data-driven approach
Source: PLoS One. 2024 Jul 5;19(7):e0305920. doi: 10.1371/journal.pone.0305920 (PMC11226107; doi:10.1371/journal.pone.0305920)

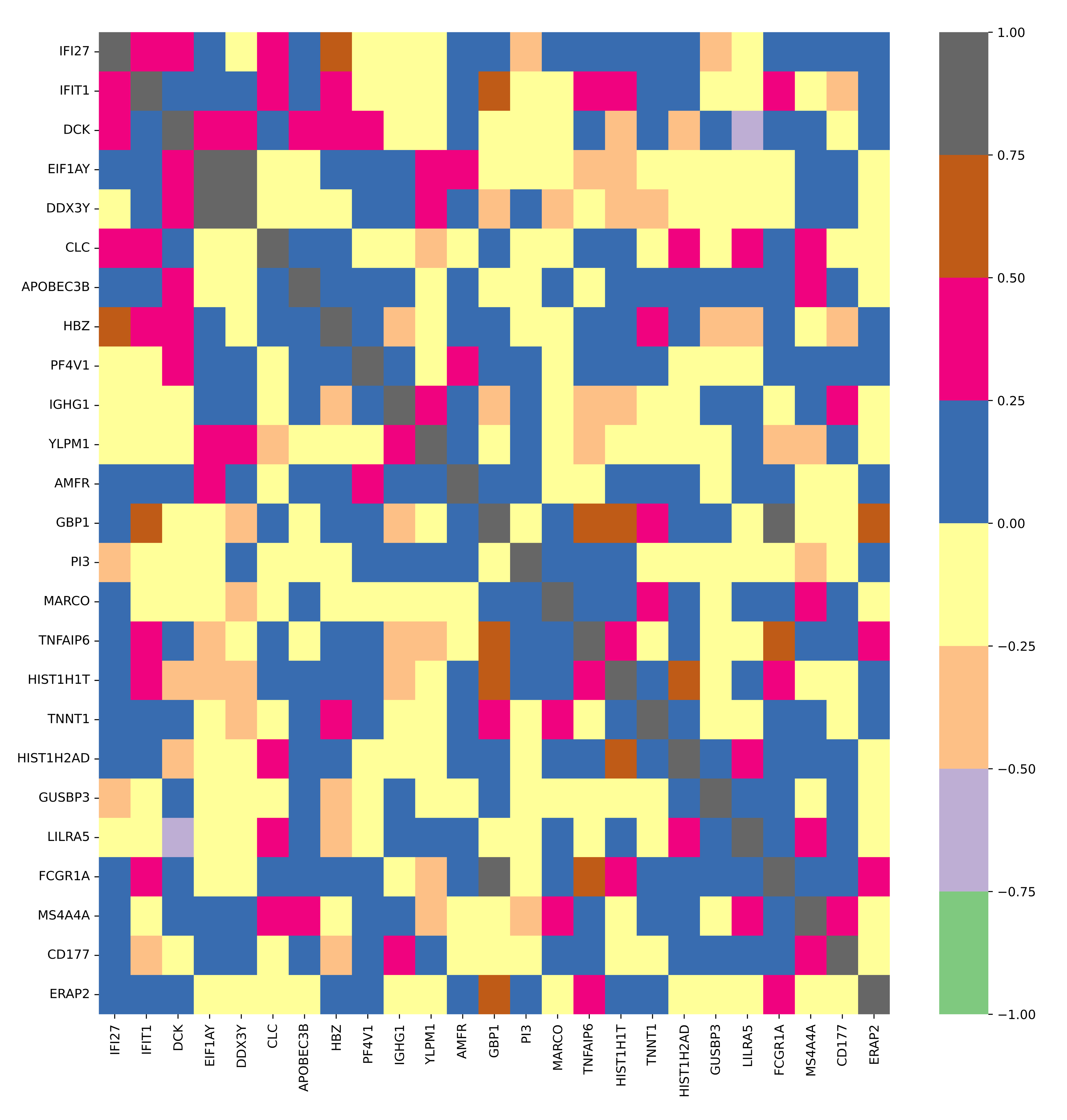

Supplement: S1 Fig — (TIF) [file pone.0305920.s001.tif]

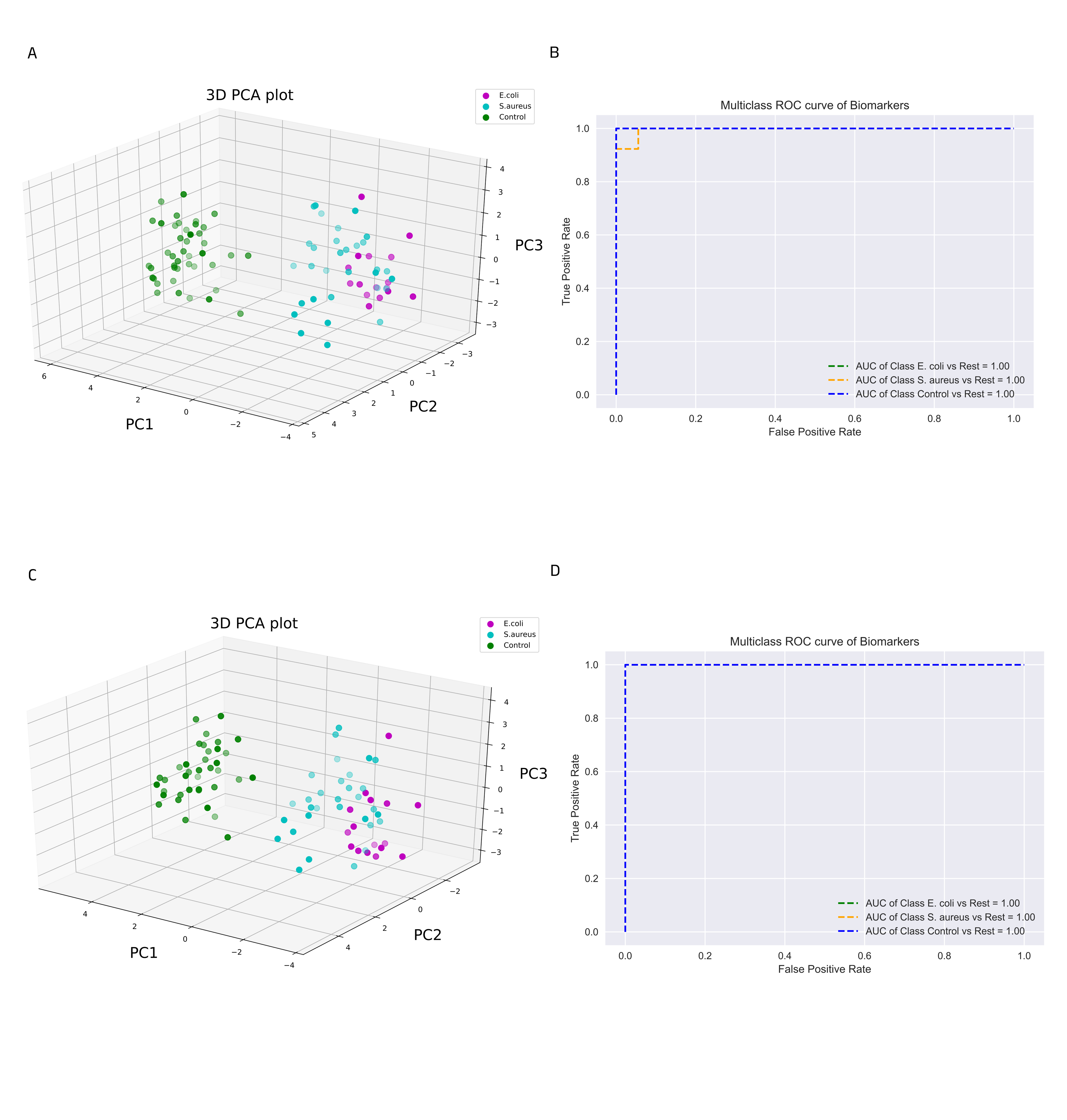

Supplement: S2 Fig — (A) Principal component analysis (PCA) plot and (B) Receiver operating characteristic (ROC) curve of age-gender balanced data (n = 151). (C) PCA plot and (D) ROC curve in group balanced data (n = 228). (TIF) [file pone.0305920.s002.tif]
