# Supplementary material for: Transcriptional markers classifying Escherichia coli and Staphylococcus aureus induced sepsis in adults: A data-driven approach
Source: PLoS One. 2024 Jul 5;19(7):e0305920. doi: 10.1371/journal.pone.0305920 (PMC11226107; doi:10.1371/journal.pone.0305920)
Supplement: S1 Table — (DOCX) [file pone.0305920.s003.docx]

| **Sample** | **Gene** | **Positive Coefficient** | **Gene** | **Negative Coefficient** |
| --- | --- | --- | --- | --- |
| **Whole dataset (n = 94)** | *IFI27* | 0.002881147 | *AMFR* | -0.003646205 |
|  | *IFIT1* | 0.038257945 | *GBP1* | -0.009361866 |
|  | *DCK* | 0.058605274 | *PI3* | -0.000103139 |
|  | *EIF1AY* | 0.00209795 | *MARCO* | -4.20E-05 |
|  | *DDX3Y* | 0.026761014 | *TNFAIP6* | -0.054268902 |
|  | *CLC* | 0.019316271 | *HIST1H1T* | -0.001788501 |
|  | *APOBEC3B* | 0.018511076 | *TNNT1* | -0.018785541 |
|  | *HBZ* | 0.008048641 | *HIST1H2AD* | -0.009471913 |
|  | *PF4V1* | 0.006544974 | *GUSBP3* | -0.026534852 |
|  | *IGHG1* | 0.039405155 | *LILRA5* | -0.025594504 |
|  | *YLPM1* | 0.037414014 | *FCGR1A* | -0.056150575 |
|  |  |  | *MS4A4A* | -0.04067223 |
|  |  |  | *CD177* | -0.06776166 |
|  |  |  | *ERAP2* | -0.002085012 |
| **Subset dataset* (n = 76)** | *RPS4Y1* | 0.021226783 | *GBP1* | -0.023531818 |
|  | *IFIT1* | 0.022298854 | *PI3* | -0.017059097 |
|  | *AGL* | 0.013882287 | *TNFAIP6* | -0.000504441 |
|  | *EIF1AY* | 0.001060983 | *GUSBP3* | -0.052094762 |
|  | *APOBEC3B* | 0.029199719 | *LILRA5* | -0.070913134 |
|  | *HBZ* | 0.001342556 | *FCGR1A* | -0.042624656 |
|  | *PF4V1* | 0.004920118 | *SLAMF7* | -0.021378698 |
|  | *RBM3* | 0.004034219 | *MS4A4A* | -0.060407356 |
|  | *IGHG1* | 0.02899031 | *CD177* | -0.088221992 |
|  | *YLPM1* | 0.031214265 |  |  |
|  | *HLA-DQA1* | 0.004637932 |  |  |
| **Includes training set of the whole dataset* | | | | |

**Table S1.**
