# Supplementary material for: Transcriptional markers classifying Escherichia coli and Staphylococcus aureus induced sepsis in adults: A data-driven approach
Source: PLoS One. 2024 Jul 5;19(7):e0305920. doi: 10.1371/journal.pone.0305920 (PMC11226107; doi:10.1371/journal.pone.0305920)
Supplement: S2 Table — (DOCX) [file pone.0305920.s004.docx]

**Table S2.**

| **Model**  **(samples, n)** | **Groups** | **Precision** | **Recall** | **f1-score** | **Support** | **PC1** | **PC2** | **PC3** |
| --- | --- | --- | --- | --- | --- | --- | --- | --- |
| **22277 genes (n=94)** |  |  |  |  |  | 21.24 | 16.10 | 10 |
| **25 genes (n=94)** | *E. coli* | 1 | 0.8 | 0.89 | 5 |  |  |  |
|  | *S. aureus* | 0.88 | 1 | 0.93 | 7 |  |  |  |
|  | Control | 1 | 1 | 1 | 7 |  |  |  |
|  | accuracy |  |  | 0.95 | 19 | 35.98 | 9.84 | 8.42 |
| **22 genes (n=94)** | *E. coli* | 1 | 0.8 | 0.89 | 5 |  |  |  |
|  | *S. aureus* | 0.88 | 1 | 0.93 | 7 |  |  |  |
|  | Control | 1 | 1 | 1 | 7 |  |  |  |
|  | accuracy |  |  | 0.95 | 19 | 38.4 | 9.73 | 6.69 |
| **25 genes (n=151)** | *E. coli* | 0.9 | 1 | 0.95 | 9 |  |  |  |
|  | *S. aureus* | 1 | 0.92 | 0.96 | 13 |  |  |  |
|  | Control | 1 | 1 | 1 | 9 |  |  |  |
|  | accuracy |  |  | 0.97 | 31 | 33.21 | 14.85 | 9.69 |
| **25 genes (n=228)** | *E. coli* | 1 | 1 | 1 | 11 |  |  |  |
|  | *S. aureus* | 1 | 1 | 1 | 19 |  |  |  |
|  | Control | 1 | 1 | 1 | 16 |  |  |  |
|  | accuracy |  |  | 1 | 46 | 35.3 | 14.31 | 10.01 |
