# Supplementary material for: Transcriptional markers classifying Escherichia coli and Staphylococcus aureus induced sepsis in adults: A data-driven approach
Source: PLoS One. 2024 Jul 5;19(7):e0305920. doi: 10.1371/journal.pone.0305920 (PMC11226107; doi:10.1371/journal.pone.0305920)
Supplement: S3 Table — (A) Categories of biological process for 25 predictive genes based on the Gene Ontology enrichment analysis (B) Categories of pathways for 25 predictive genes based on Reactome pathway analysis. (DOCX) [file pone.0305920.s005.docx]

**Table S3A.**

| **Ontology Description** | **ID** | **Gene no.** | **Fraction of total genes %** | **fold Enrichment** | ***p*-value** | **FDR** | **Gene name** |
| --- | --- | --- | --- | --- | --- | --- | --- |
| **Immune system process** | GO:0002376 | 13 | 52 | 4.56 | 8.75E-07 | 2.73E-03 | *PI3/IFIT1/CD177/ LILRA5/IFI27/ MARCO/FCGR1A/ APOBEC3B/GBP2/PF4V1/ERAP2/IGHG2/ CLC* |
| **Defense response** | GO:0006952 | 12 | 48 | 6.71 | 4.56E-08 | 7.11E-04 | *PI3/IFIT1/CD177/ LILRA5/IFI27/ MARCO/FCGR1A/ APOBEC3B/GBP2/ PF4V1/TNFAIP6/ IGHG1* |
| **Immune response** | GO:0006955 | 12 | 48 | 5.85 | 2.04E-07 | 1.06E-03 | *PI3/IFIT1/CD177/ LILRA5/IFI27/ MARCO/FCGR1A/ APOBEC3B/GBP2/ PF4V1/ERAP2/ IGHG2* |
| **Defense response to other organism** | GO:0098542 | 10 | 40 | 7.94 | 1.92E-07 | 1.50E-03 | *PI3/IFIT1/CD177/ LILRA5/IFI27/ MARCO/FCGR1A/ APOBEC3B/GBP2/ PF4V1* |
| **Response to other organism** | GO:0051707 | 10 | 40 | 5.81 | 3.29E-06 | 8.56E-03 | *PI3/IFIT1/CD177/ LILRA5/IFI27/ MARCO/FCGR1A/ APOBEC3B/GBP2/ PF4V1* |
| **Response to external biotic stimulus** | GO:0043207 | 10 | 40 | 5.8 | 3.36E-06 | 7.49E-03 | *PI3/IFIT1/CD177/ LILRA5/IFI27/ MARCO/FCGR1A/ APOBEC3B/GBP2/ PF4V1* |
| **Response to biotic stimulus** | GO:0009607 | 10 | 40 | 5.61 | 4.48E-06 | 8.75E-03 | *PI3/IFIT1/CD177/ LILRA5/IFI27/ MARCO/FCGR1A/APOBEC3B/GBP2/ PF4V1* |
| **Biological process involved in interspecies interaction between organisms** | GO:0044419 | 10 | 40 | 5.14 | 9.85E-06 | 1.71E-02 | *PI3/IFIT1/CD177/ LILRA5/IFI27/ MARCO/FCGR1A/ APOBEC3B/GBP2/ PF4V1* |
| **Innate immune response** | GO:0045087 | 9 | 36 | 9.48 | 2.16E-07 | 8.41E-04 | *PI3/IFIT1/CD177/ LILRA5/IFI27/ MARCO/FCGR1A/ APOBEC3B/GBP1* |
| **Type II hypersensitivity** | GO:0002445 | 2 | 8 | > 100 | 2.29E-05 | 3.57E-02 | *FCGR1A/IGHG1* |
| **Type IIa hypersensitivity** | GO:0001794 | 2 | 8 | > 100 | 2.29E-05 | 3.25E-02 | *FCGR1A/IGHG2* |
| **Antibody-dependent cellular cytotoxicity** | GO:0001788 | 2 | 8 | > 100 | 2.29E-05 | 2.97E-02 | *FCGR1A/IGHG3* |

**Table S3B.**

| **Reactome pathways** | **Gene no.** | **Fraction of total genes**  **%** | **fold Enrichment** | ***p*-value** | **Gene name** |
| --- | --- | --- | --- | --- | --- |
| **Immune System** | 11 | 44 | 4.24 | 1.85E-05 | *PI3/IFIT1/ERAP2/ERAP2/CD177/CLC/LILRA5/IFI27/FCGR1A/GBP1/IGHG1* |
| **Cytokine Signaling in Immune system** | 6 | 24 | 6.82 | 1.98E-04 | *IFIT1/CLC/IFI27/FCGR1A/GBP1/IGHG1* |
| **Innate Immune System** | 5 | 20 | 3.58 | 1.15E-02 | *PI3/TNFAIP6/CD177/ FCGR1A/ IGHG1* |
| **Interferon Signaling** | 4 | 16 | 16.08 | 1.11E-04 | *IFIT1/IFI27/FCGR1A/ GBP1* |
| **Immunoregulatory interactions between a Lymphoid and a non-Lymphoid cell** | 2 | 8 | 8.04 | 2.59E-02 | *LILRA5/FCGR1A* |
| **Common Pathway of Fibrin Clot Formation** | 2 | 8 | 71.99 | 4.15E-04 | *CD177/PF4V1* |
| **Formation of Fibrin Clot Clotting Cascade** | 2 | 8 | 40.61 | 1.22E-03 | *CD177/PF4V1* |
| **Interferon alpha/beta signaling** | 2 | 8 | 23.29 | 3.50E-03 | *IFIT1/IFI27* |
| **FCGR activation** | 2 | 8 | 21.4 | 4.12E-03 | *FCGR1A/IGHG1* |
| **Role of phospholipids in phagocytosis** | 2 | 8 | 18 | 5.72E-03 | *FCGR1A/IGHG1* |
| **Interferon gamma signaling** | 2 | 8 | 17.4 | 6.10E-03 | *FCGR1A/GBP1* |
| **FCGR3A-mediated IL10 synthesis** | 2 | 8 | 15.84 | 7.29E-03 | *FCGR1A/IGHG1* |
| **Regulation of actin dynamics for phagocytic cup formation** | 2 | 8 | 12.88 | 1.08E-02 | *FCGR1A/IGHG1* |
| **Fcgamma receptor (FCGR) dependent phagocytosis** | 2 | 8 | 10.77 | 1.51E-02 | *FCGR1A/IGHG1* |
| **Cell surface interactions at the vascular wall** | 2 | 8 | 8.04 | 2.59E-02 | *CD177/PF4V1* |
| **Leishmania parasite growth and survival** | 2 | 8 | 6.92 | 3.41E-02 | *FCGR1A/IGHG1* |
| **Anti-inflammatory response favouring Leishmania parasite infection** | 2 | 8 | 6.92 | 3.41E-02 | *FCGR1A/IGHG1* |
| **mRNA Editing: C to U Conversion** | 1 | 4 | 98.99 | 1.13E-02 | *APOBEC3B* |
| **Formation of the Editosome** | 1 | 4 | 98.99 | 1.13E-02 | *APOBEC3B* |
| **mRNA Editing** | 1 | 4 | 79.19 | 1.38E-02 | *APOBEC3B* |
| **IL-6-type cytokine receptor ligand interactions** | 1 | 4 | 46.58 | 2.25E-02 | *CLC* |
| **Scavenging by Class A Receptors** | 1 | 4 | 41.68 | 2.49E-02 | *MARCO* |
| **ER Quality Control Compartment (ERQC)** | 1 | 4 | 37.71 | 2.74E-02 | *AMFR* |
| **Interleukin-6 family signaling** | 1 | 4 | 33 | 3.11E-02 | *CLC* |
| **Calnexin/calreticulin cycle** | 1 | 4 | 30.46 | 3.35E-02 | *AMFR* |
| **Antigen Presentation: Folding, assembly and peptide loading of class I MHC** | 1 | 4 | 30.46 | 3.35E-02 | *ERAP2* |
| **Metalloprotease DUBs** | 1 | 4 | 25.54 | 3.96E-02 | *H2AC7* |
| **RNA Polymerase I Promoter Opening** | 1 | 4 | 24.75 | 4.08E-02 | *H2AC7* |
| **Packaging Of Telomere Ends** | 1 | 4 | 24 | 4.20E-02 | *H2AC7* |
| **DNA methylation** | 1 | 4 | 23.29 | 4.32E-02 | *H2AC7* |
| **N-glycan trimming in the ER and Calnexin/Calreticulin cycle** | 1 | 4 | 22.63 | 4.45E-02 | *AMFR* |
| **Striated Muscle Contraction** | 1 | 4 | 22 | 4.57E-02 | *TNNT1* |
| **Depurination** | 1 | 4 | 22 | 4.57E-02 | *H2AC7* |
| **Activated PKN1 stimulates transcription of AR androgen receptor) regulated genes KLK2 and KLK3** | 1 | 4 | 22 | 4.57E-02 | *H2AC7* |
| **Cleavage of the damaged purine** | 1 | 4 | 22 | 4.57E-02 | *H2AC7* |
| **Pyrimidine salvage** | 1 | 4 | 71.99 | 1.50E-02 | *DCK* |
| **Nucleotide salvage** | 1 | 4 | 34.43 | 2.99E-02 | *DCK* |
| **Purine salvage** | 1 | 4 | 60.91 | 1.75E-02 | *DCK* |
| **Recognition and association of DNA glycosylase with site containing an affected purine** | 1 | 4 | 22 | 4.57E-02 | *H2AC7* |
| **SIRT1 negatively regulates rRNA expression** | 1 | 4 | 21.4 | 4.69E-02 | *H2AC7* |
| **Assembly of the ORC complex at the origin of replication** | 1 | 4 | 20.84 | 4.81E-02 | *H2AC7* |
