# Supplementary material for: Transcriptional markers classifying Escherichia coli and Staphylococcus aureus induced sepsis in adults: A data-driven approach
Source: PLoS One. 2024 Jul 5;19(7):e0305920. doi: 10.1371/journal.pone.0305920 (PMC11226107; doi:10.1371/journal.pone.0305920)
Supplement: S1 File — (DOCX) [file pone.0305920.s006.docx]

**Supporting information**

**S1 File. Models and upsampling techniques performances**

*Model performance:*

To determine the best model for feature selection, we employed Ridge regression with a tuned lambda value of 0.99 as one of our methodologies. This approach resulted in selection of all genes with an MSE of 0.14. However, as Ridge regression inherently lacks feature selection capabilities, we employed an indirect method to identify significant genes. This involved analyzing coefficient distributions and establishing a threshold at 0.002 (Figure 1), which allowed us to identify 181 genes.


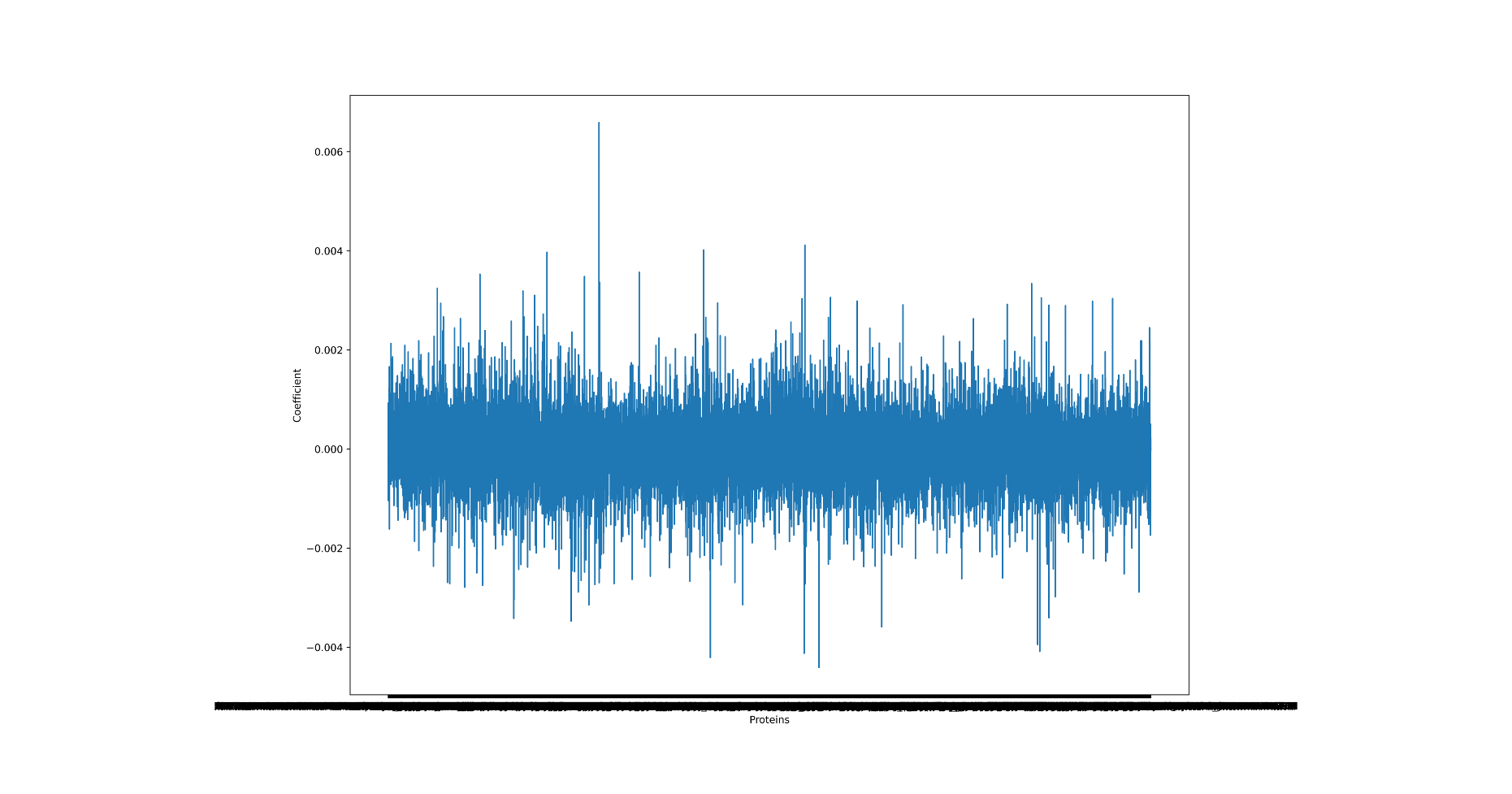


***Figure 1.*** Ridge regression coefficient distribution.

Subsequent evaluation using logistic regression on 94 samples showed perfect AUC values for each sample group. Nevertheless, high performances are observed both with and without determining the threshold. Given the small sample size relative to the number of covariates, this could lead to inflated performance metrics and overfitting (1).

We also employed Elastic Net regression with a tuned lambda value of 0.11, resulting in an MSE of 0.19 while selecting 32 genes. Although the performance was comparable to Lasso regression (MSE 0.20), the number of covariates increased to 32 genes from 25. Lasso is the putative option when aiming to restrict the number of covariates, which aligns with the objective of this study.

*Upsampling performance*:

We addressed the issue of imbalanced data by employing two upsampling methods: SMOTE and random upsampling of minority groups. SMOTE resulted in a dataset of 129 samples, while random upsampling produced datasets of 151 and 228 samples. Following the application of Ridge, Elastic Net and Lasso regression models and subsequent parameter tuning, both models exhibited a lambda value of 0, resulting in the retention of all genes. The MSE varied across upsampling strategies: for SMOTE, Lasso regression and Elastic Net exhibited an MSE of 0.13, whereas Ridge regression yielded an MSE of 2.02. Notably, random upsampling of minority groups consistently yielding lower MSE values compared to SMOTE (Table 1).

***Table 1*.** Comparison of regression model performance with different upsampling techniques.

| **Model** | **Model** **parameters** | **SMOTE** | **Random** **upsampling** | |
| --- | --- | --- | --- | --- |
|  |  | n=129 | n=151 | n=228 |
| **Ridge** | Alpha | 0 | 0 | 0 |
|  | Selected genes (n) | 22 277 | 22 277 | 22 277 |
|  | MSE | 2.02 | 0.03 | 0.02 |
| **Elastic Net** | Alpha | 0 | 0 | 0 |
|  | Selected genes (n) | 22 277 | 22 277 | 22 277 |
|  | MSE | 0.13 | 0.07 | 0.04 |
| **Lasso** | Alpha | 0 | 0 | 0 |
|  | Selected genes (n) | 22 277 | 22 277 | 22 277 |
|  | MSE | 0.13 | 0.07 | 0.04 |

**References**

1. Hastie T, Tibshirani R, Friedman J. The elements of statistical learning: data mining, inference, and prediction. 2nd ed ed. Statistics SSi, editor: New York City: Springer; 2009.
