# Supplementary material for: Transcriptional markers classifying Escherichia coli and Staphylococcus aureus induced sepsis in adults: A data-driven approach
Source: PLoS One. 2024 Jul 5;19(7):e0305920. doi: 10.1371/journal.pone.0305920 (PMC11226107; doi:10.1371/journal.pone.0305920)
Supplement: S2 File — (DOCX) [file pone.0305920.s007.docx]

**Supporting information**

**S2 File. Sample distribution pattern before feature selection**

To illustrate the distribution of samples from individuals with *E. coli*- and *S. aureus*-induced sepsis, as well as those from healthy controls, across a multidimensional space, we employed unsupervised learning via PCA, as depicted in Figure 1 and S3 Table. The PCA plot revealed a clear distinction between healthy controls and the two patient groups. However, no detectable separation was observed among the samples from individuals with *E. coli*- and *S. aureus*-induced sepsis.


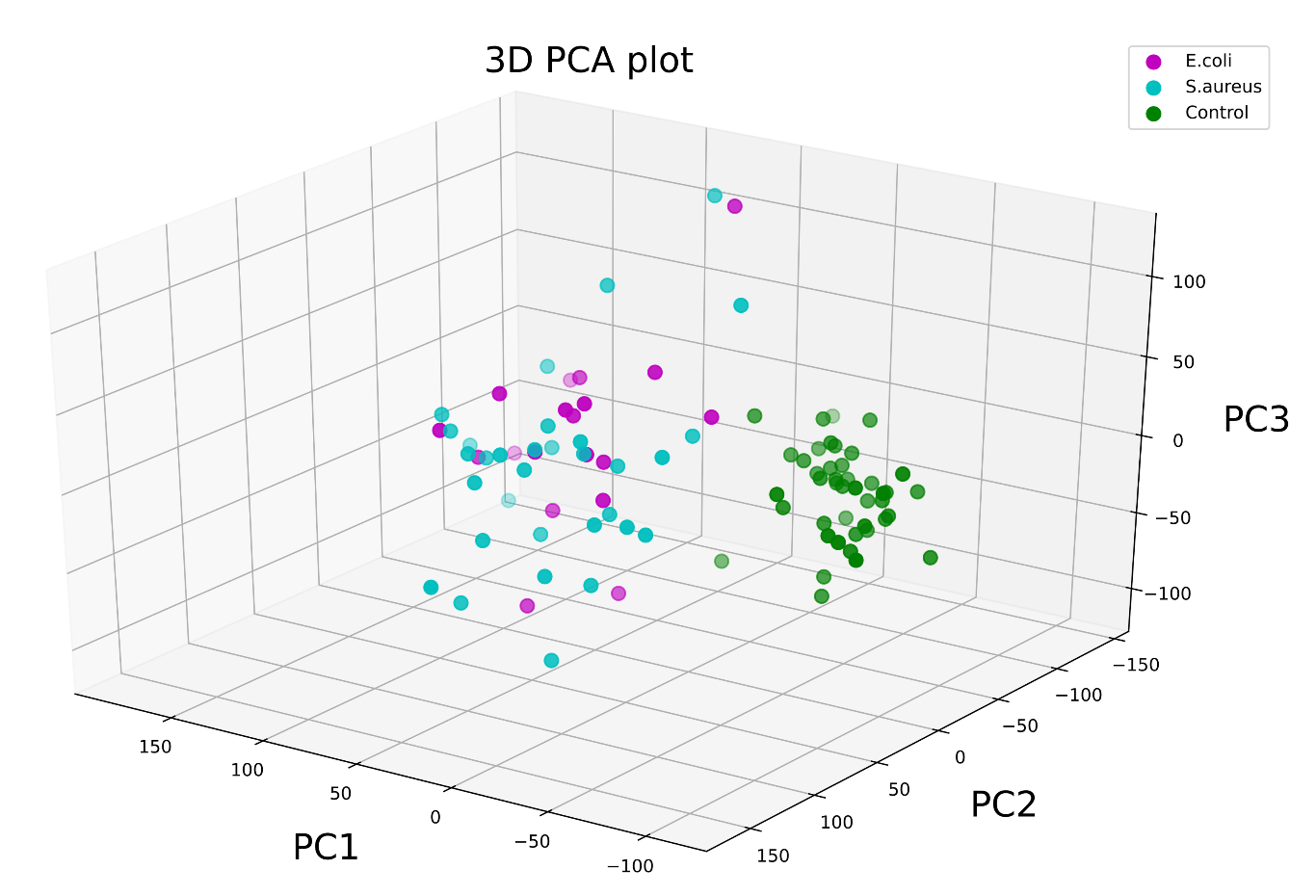


***Figure 1*.** Distribution of sample from participants.

Principal Component Analysis (PCA) plot of the distribution of samples from individuals with *E. coli*- and *S. aureus*-induced sepsis, and healthy controls (*E. coli*; purple, *S. aureus*; light blue, healthy control; green). Healthy controls exhibit a differentiation from sepsis patients, with no distinct separation between the two patient groups.
