# Supplementary material for: Transcriptional markers classifying Escherichia coli and Staphylococcus aureus induced sepsis in adults: A data-driven approach
Source: PLoS One. 2024 Jul 5;19(7):e0305920. doi: 10.1371/journal.pone.0305920 (PMC11226107; doi:10.1371/journal.pone.0305920)
Supplement: Attachment — Submitted filename: Response to Reviewers.docx [file pone.0305920.s008.docx]

Dear Editor,

Thanks very much for constructive comments and recommendations. We have addressed all the raised issues and updated the manuscript accordingly. Here are point-to-point responses to reviewers’ comments based on the ‘Revised Manuscript with Track Changes’ file.

Reviewer #1:

The methods used by the authors are not between the most advanced available now. For example, the LASSO method used for feature extraction has to compared with other methods such as RIDGE and Elastic net, or with methods for dimensionality reduction.

Finally, the numerosity of the data is not so high and the upsampling procedure used by the authors is not convincing. I would prefer to see the use of synthetic data for data augmentation.

We updated the manuscript to include a comparison with Ridge regression, Elastic Net, and oversampling using the synthetic technique SMOTE.

1. One subsection is added to Materials and Methods section (Lines 147-167)
2. One supporting file added as S1_File

We have already utilized the dimensionality reduction method Principal Component Analysis (PCA) throughout the study, as stated in lines 168-169 and mentioned in several subsections including ‘Classification analysis of three groups of participants’ and ‘Fig 1’, ‘Internal validation of selected predictive genes’ and ‘Fig 2’, ‘Stability analysis of predictive genes’, ‘S2 Fig’ and ‘S2 Table’. We also updated manuscript by including a supporting information dedicated to PCA **before** feature selection as S2_File.

Reviewer #2:

Lines 98-99: Homo sapiens should be italicized.

We have changed ‘Homo sapiens’ to ‘*Homo sapiens’*.

(Lines 128-129)

Line 111: Which version of R? The reference is missing.

We have added the version and reference.

(Line 141)

Lines 170-173: Illumina: the distribution is missing.

We have added the distribution.

(Lines 222 and 225)

Line 175: The citation for R is missing.

We have added both versions to beadarray package and added the reference for R.

(Line 227)

Line 191: Cytoscape software. Version? Reference?

We have added both version and reference.

(Line 243)

Line 205: Why is a non-parametric test (Mann-Whitney test) used first and then a parametric test (Pearson correlation)? Would it be better to use Spearman correlation?

We defined criteria for normal distribution, considering skewness and kurtosis, based on the recommendation of Hair et al (2010) [1] and Byrne (2010) [2]. This criterion justifies the methods that we have used. We have clarified this criterion by adding a sentence to the text and updating the sentence.

(Lines 301-303)

We utilized the Pearson correlation coefficient as a descriptive statistic to measure the strength and direction of the relationship between variables. The parametric aspect of the Pearson correlation arises when conducting hypothesis tests to determine if the observed correlation is significantly different from zero.

Line 325: STRING. Citation?

We have added the reference.

(Lines 379 and 243)

Line 537: "doi" is repeated twice.

We have removed the extra ‘doi’.

(Line 605)

In general, would it be possible to increase the resolution of the images (including supplementary ones)? They appear slightly grainy.

We updated all images including supplementary.

Reviewer #3:

I read the manuscript with sincere curiosity. The topic of the study is certainly since the introduction of new methodologies for sepsis prediction, and consequently its treatment, is critical. I do not have any major revision to recommend, just a few details that can be improved.
Line 5-6, 33: Never mind specifying the abbreviation given to the name of the bacteria, it is in common use.

We have removed abbreviations.

(Lines 30-31 and 58)

Line 57: Do you have considered the costs analysis for the introduction of the microarray in the diagnostic workflow? May the author should add a paragraph about.

We updated the text.

(Lines 83-88)

Line 98-99: Homo sapiens in italics.

We have changed ‘Homo sapiens’ to ‘*Homo sapiens’.*

(Lines 128-129)

Line 180: what do you meant with “multiple synonyms”?

In the context of our study, 'multiple synonymous names' refer to genes that are known by more than one identifier or name within the microarray dataset. For instance, some genes listed among the 25-gene dataset were labeled by several names, which may indicate their official symbol, alternative symbols, aliases, or historical names, separated by the '///' sign (i.e., ‘ABCD///EFG///HIJK’). We updated the name to ‘multiple annotations’.

(Line 233)

All over the manuscript: it should be appropriate if the author enter in brackets: the supplier, supplier’s head office and country following each kit and instruments included in the study.

We have added the distribution.

(Lines 222 and 225)

1.	Hair J, J F, Black JW, Babin BJ, Anderson ER. Multivariate Data Analysis. Seventh ed: Edinburgh: Pearson Education Limited; 2010.

2.	Byrne BM. Structural equation modeling with AMOS: Basic concepts, applications, and programming: New York: Routledge; 2010.
